# Cameron Lesions: Endoscopic Characteristics, Clinical Profile, and Therapeutic Implications—A Retrospective Study of 43 Patients

**DOI:** 10.3390/diagnostics16183051

**Published:** 2026-09-20

**Authors:** Viorel Istrate, Alex-Claudiu Moraru, Sergiu Ungureanu, Bogdan Istrate, Florin Achim, Adrian Constantin, Alexandru Rotariu, Anthony Rasuceanu, Bogdan Socea, Cristian Rosianu, Eugenia Panaitescu, Dragos Predescu

**Affiliations:** 1 Faculty of Medicine, Nicolae Testemițanu State University of Medicine and Pharmacy, MD-2004 Chisinau, Moldova; viorelistrate2@gmail.com (V.I.); sergiu.ungureanu@usmf.md (S.U.); bogdanistrate2@gmail.com (B.I.); 2 Faculty of Medicine, Carol Davila University of Medicine and Pharmacy, 050474 Bucharest, Romania; alex.claudiu.moraru@gmail.com (A.-C.M.); alexandru.rotariu95@yahoo.com (A.R.); rasuceanu.anthony@gmail.com (A.R.); bogdan.socea@umfcd.ro (B.S.); e.panaitescu@yahoo.com (E.P.); drpredescu@yahoo.com (D.P.); 3 Department of General and Esophageal Surgery, Sf. Maria Clinical Hospital, 011192 Bucharest, Romania; 4 Department of Surgery, Sf. Pantelimon Clinical Emergency Hospital, 021659 Bucharest, Romania; 5 Department of Gastroenterology, Sf. Maria Clinical Hospital, 011192 Bucharest, Romania; rosianu_cristian@yahoo.com; 6 Intensive Computing and Computational Medicine Centre (CCIMC), Medical Informatics and Biostatistics Department, Faculty of Medicine, Carol Davila University of Medicine and Pharmacy, 050474 Bucharest, Romania

**Keywords:** Cameron lesions, hiatal hernia, herniated gastric ulcer, iron-deficiency anemia, upper gastrointestinal bleeding, digestive endoscopy, fundoplication

## Abstract

**Background and Objectives:** Cameron lesions are linear gastric erosions or ulcers that develop at the level of the diaphragmatic constriction within a hiatal hernia. They represent an underrecognized cause of chronic iron-deficiency anemia and upper gastrointestinal bleeding. This study aimed to characterize the demographic, clinical, endoscopic, and therapeutic features of Cameron lesions diagnosed at a tertiary referral digestive endoscopy center in the Republic of Moldova. **Methods:** This was a retrospective single-center study from Republic of Moldova conducted over a 9-year period (2017–2026). Forty-three consecutive patients with endoscopically confirmed Cameron lesions were included. All examinations were performed by a single experienced endoscopist using high-definition endoscopy systems (Olympus EVIS Exera III and Olympus EVIS X1). Demographic characteristics, hiatal hernia type and size, clinical presentation, endoscopic findings, bleeding stigmata, *Helicobacter pylori* status, and postoperative outcomes were analyzed. **Results:** The mean age of the patients was 60.4 years (range, 31–83 years), with a female predominance (65.1%; 28 women and 15 men). The distribution of hiatal hernia types was as follows: type I (axial/sliding), 32.6% (*n* = 14); type II (paraesophageal/rolling), 34.8% (*n* = 15); and type III (mixed), 32.6% (*n* = 14). Cameron lesions developed after failed fundoplication in three patients (6.9%) and were identified in fixed (39.5%), completely reducible (16.3%), and partially reducible (mixed) hernias (44.2%). The main indications for upper gastrointestinal endoscopy were GERD symptoms (53.4%), iron-deficiency anemia (20.9%), prophylactic or postoperative surveillance (18.6%), and overt upper gastrointestinal bleeding (7.0%). Active Cameron lesions were identified in 55.8% of patients, whereas cicatricial and mixed lesions accounted for 30.2% and 13.9%, respectively. Bleeding stigmata were classified according to Forrest as Ia (2.3%), Ib (18.6%), IIa (4.65%), IIb (11.6%), IIc (7.0%), and III (55.81%). Nine patients (20.9%) were identified with active bleeding. Importantly, Cameron lesions were also identified in small hiatal hernias (<3 cm) and in patients with recurrent hiatal hernia after failed fundoplication. *Helicobacter pylori* infection was detected in 25.6% (*n* = 11) of patients. **Conclusions:** This study confirms the well-established association between Cameron lesions, female sex, hiatal hernias, and gastrointestinal bleeding while providing several novel observations, including their occurrence in small hiatal hernias, diagnosis in relatively young adults, and recurrence following failed fundoplication. The findings also highlight the potential contribution of the reducible component of hiatal hernias to lesion development and emphasize the importance of meticulous inspection of the hernia sac during every upper gastrointestinal endoscopic examination.

## 1. Introduction

Cameron lesions, named after Dr. Adrian J. Cameron, a gastroenterologist at the Mayo Clinic, are linear superficial gastric erosions or ulcers located on the crests of gastric mucosal folds at the level of the diaphragmatic constriction, typically occurring in association with hiatal hernias. This entity was first systematically described in 1986 by Cameron and Higgins in a prospective controlled study, which established the association between linear gastric erosions and chronic iron-deficiency anemia in patients with large hiatal hernias [1,2].

The clinical importance of Cameron lesions stems from several factors. First, they represent a common but frequently overlooked cause of chronic iron-deficiency anemia without an obvious source of bleeding, often leading to extensive diagnostic investigations and delayed or incomplete treatment [3,4,5,6,7,8,9]. A systematic review by Zullo et al., published in 2018, reported that 69% of patients with confirmed Cameron lesions had undergone at least one previous upper gastrointestinal endoscopy in which the lesions had not been recognized [10]. Second, although less common, Cameron lesions may also present with acute upper gastrointestinal bleeding, occasionally resulting in life-threatening hemorrhage [11,12,13,14,15]. Third, their distinctive pathophysiological mechanism, which is believed to be predominantly mechanical and ischemic rather than acid-peptic, distinguishes Cameron lesions from conventional peptic ulcer disease and has important therapeutic implications [16,17,18].

Despite four decades of increasing awareness, Cameron lesions remain an underrecognized entity in routine gastroenterological practice [19,20]. Their underdiagnosis is attributable to several factors, including the absence of a standardized classification system, variability in endoscopic appearance, and their characteristic location within the hiatal hernia sac [21,22,23]. The available evidence consists predominantly of case reports, small case series, and a limited number of systematic reviews, whereas randomized controlled studies are lacking [24,25,26]. To date, no epidemiological or clinical data on Cameron lesions have been published from the Republic of Moldova. To the best of our knowledge, the present study represents the first national case series dedicated exclusively to this condition and summarizes the experience of a single expert endoscopist over a nine-year period.

The objectives of this study were to: (1) characterize the demographic profile of patients diagnosed with Cameron lesions at a tertiary referral endoscopy center; (2) analyze the distribution of the associated hiatal hernia types, including hernia size and reducibility; (3) describe the clinical presentation and endoscopic characteristics of Cameron lesions; (4) evaluate postoperative outcomes in patients with a history of fundoplication; and (5) compare the findings of the present study with those reported in the international literature.

## 2. Materials and Methods

### 2.1. Study Design and Population

This retrospective, analytical, single-center study was based on the clinical activity of a single expert endoscopist and was conducted over a 9-year period (2017–2026). Consecutive patients with an endoscopic diagnosis of Cameron lesions were included. Cameron lesions were defined as linear gastric erosions or ulcers located within the hernia sac, at the hernia neck, or at the level of the diaphragmatic impression. Patients with post-Cameron scar sequelae (Sakita–Miwa stages S1–S2) in the presence of a hiatal hernia were also included.

### 2.2. Inclusion and Exclusion Criteria

The inclusion criteria were as follows: (1) endoscopically confirmed active Cameron lesions or post-Cameron scar sequelae; (2) endoscopic evidence of a hiatal hernia during the same examination; and (3) complete endoscopic documentation in the institutional database. The exclusion criteria were as follows: (1) patients described as having a “Cameron precursor” or being “at risk of Cameron lesions” without endoscopic confirmation; (2) duplicate records; and (3) incomplete clinical or endoscopic documentation. Patients who underwent multiple endoscopic examinations were included only once, whereas follow-up examinations were analyzed separately.

### 2.3. Data Collection

Clinical and endoscopic data were extracted from the institutional electronic endoscopy database. The following variables were collected: age, sex, indication for endoscopy, hiatal hernia type (types I–IV), Cameron lesion status (active or cicatricial), bleeding stigmata according to the Forrest classification, associated upper gastrointestinal pathology (reflux esophagitis, Barrett’s esophagus, gastritis, and duodenitis), *Helicobacter pylori* status determined by the rapid urease test, history of fundoplication, and postoperative outcomes. All patients included in the study signed an informed consent form at the time of their clinical and endoscopic evaluations, which explicitly allows the anonymized use of their medical data for future research purposes. The study was conducted in accordance with the Declaration of Helsinki and institutional data protection regulations.

### 2.4. Hiatal Hernia Characteristics and Measurement Protocol

Hiatal hernia characteristics were analyzed using prospectively recorded endoscopic data collected over the 9-year study period. Between March 2017 and March 2026, a total of 14,685 upper gastrointestinal endoscopic examinations were performed, of which 1824 (12.4%) documented the presence of a hiatal hernia. All examinations were performed by a single experienced endoscopist using high-definition endoscopy systems (Olympus EVIS Exera III and Olympus EVIS X1, Olympus, Tokyo, Japan). Hiatal hernias were classified according to the standard anatomical classification into type I (sliding), type II (paraesophageal), type III (mixed), and type IV (involving additional abdominal organs). Hiatal hernia size and reducibility were systematically recorded. Hernias were classified as small (<3 cm), medium (3.1–5.0 cm), or large (>5 cm). The competence of the esophagogastric junction (EGJ) was routinely assessed using the Hill classification (grades I–IV). To quantify the volume of the hiatal hernia, the hernia sac was geometrically modelled as an ellipsoid. Three orthogonal dimensions were measured for each case: the antero-posterior diameter, the transverse diameter and the distance between cardia and hiatal orifice as cranio-caudal height. A standardized measurement protocol was used. Lesions < 1 cm were measured using open biopsy forceps (7.5 mm) or estimated by comparison with the diameter of closed biopsy forceps (2.5 mm), whereas lesions ≥ 1 cm were measured using a graduated endoscopic measuring probe.

### 2.5. Statistical Analysis

Continuous variables are presented as mean ± standard deviation (SD) or median with interquartile range (IQR), as appropriate according to data distribution. Categorical variables are expressed as absolute frequencies and percentages.

Statistical analyses were performed using IBM SPSS Statistics version 23.0 (IBM Corp., Armonk, NY, USA) and Microsoft Excel (Redmond, WA, USA). The distribution of continuous variables was assessed for normality and homogeneity of variances using the Shapiro–Wilk and Levene tests, respectively.

Comparisons between two normally distributed groups were performed using Student’s *t*-test, whereas the Mann–Whitney *U* test was used for non-normally distributed data. Comparisons involving more than two non-normally distributed groups were performed using the Kruskal–Wallis test. Associations between categorical variables were analyzed using the likelihood ratio χ^2^ test. Correlations between continuous variables were assessed using Pearson’s correlation coefficient for approximately normally distributed data and Spearman’s rank correlation coefficient for ordinal or non-normally distributed variables.

Univariate linear regression analyses were performed to identify factors associated with the maximum size of Cameron lesions. Although multivariable linear regression was explored, no statistically adequate multivariable model could be established because of the limited sample size and data characteristics. A two-sided *p* value < 0.05 was considered statistically significant.

## 3. Results

### 3.1. Demographic Characteristics

**Figure 1:**
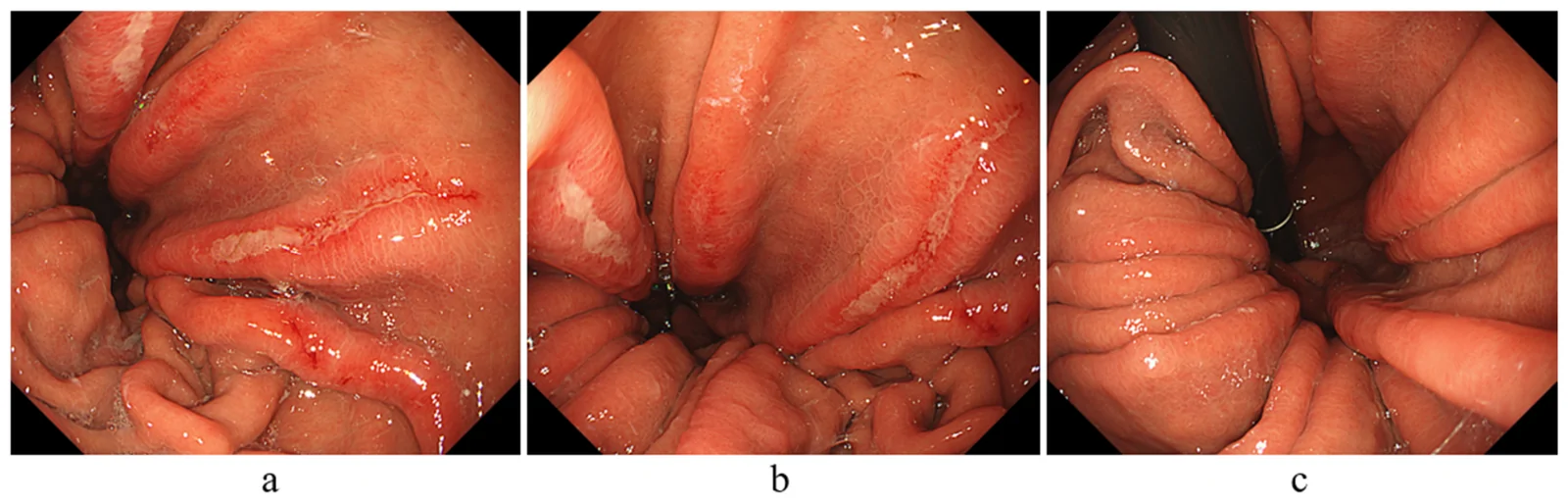
Cameron lesions (*n* = 4; 15–30 mm; Sakita–Miwa A1, Forrest Ib) associated with a large mixed type III hiatal hernia: (**a**,**b**) antegrade endoscopic view, (**c**) retroflexed view from the stomach.

Between March 2017 and March 2026, a total of 14,685 upper gastrointestinal endoscopic examinations were performed, of which 1824 (12.4%) documented the presence of a hiatal hernia. Cameron ulcers were endoscopically confirmed in 43 patients (2.35%) with hiatal hernia (Figure 1). The incidence of Cameron lesions relative to the total number of endoscopies was 0.29%.

Forty patients (93.0%) had at least one documented follow-up endoscopic examination. The demographic characteristics of the study cohort are summarized in Table 1.

The mean age was 60.4 ± 12.7 years (range, 31–83 years). The cohort comprised 28 women (65.1%) and 15 men (34.9%), corresponding to a female-to-male ratio of 1.87:1. Three patients (7.0%) had previously undergone anti-reflux fundoplication and subsequently developed recurrent hiatal hernia associated with Cameron lesions.

The youngest patient was a 31-year-old man, whereas the oldest was an 83-year-old woman. Their detailed clinical characteristics are presented in the section Illustrative Clinical Cases (Section 3.9).

Statistical analysis demonstrated that the maximum Cameron lesion size was significantly greater in women than in men (21.7 ± 6.8 mm vs. 17.1 ± 6.2 mm, Student’s *t*-test, *p* = 0.0368). In addition, a weak positive correlation was observed between patient age and maximum lesion size (Pearson’s *r* = 0.320, *p* = 0.0366), indicating a tendency toward larger lesions in older patients.

### 3.2. Hiatal Hernia Characteristics

According to hernia size, 1006 hernias (55.2%) were small, 712 (39.0%) were medium-sized, and 106 (5.8%) were large. Hernias were further categorized according to reducibility as completely reducible (664; 36.4%) or fixed (1160; 63.6%). These parameters were analyzed to evaluate their association with the occurrence of Cameron lesions.

Distribution by Hill Grade. Advanced EGJ incompetence predominated, with Hill grade IV observed in 1390 patients (76.2%), followed by Hill grade III in 298 (16.3%) and Hill grade II in 136 (7.5%). No patients were classified as Hill grade I.

#### 3.2.1. Anatomical Classification of Hiatal Hernias

The distribution according to anatomical classification was as follows: type I, 1386 cases (76.0%); type II, 221 (12.1%); type III, 213 (11.7%); and type IV, 4 (0.2%).

The anatomical distribution of hiatal hernias among patients with Cameron lesions is presented in Table 2. Type II (paraesophageal/rolling) hiatal hernias were the most frequent, accounting for 15 patients (34.8%), followed by type I (axial/sliding) and type III (mixed) hernias, each observed in 14 patients (32.6%). No type IV hiatal hernias were identified.

Statistical analysis demonstrated a significant association between anatomical hiatal hernia type and hernia size (Likelihood Ratio χ^2^, *p* = 0.000738). Large hiatal hernias were predominantly type III (71.4%), medium-sized hernias were mainly type II (73.3%), whereas small hiatal hernias were most frequently type I (50.0%) (Table 2).

#### 3.2.2. Esophagogastric Junction Competence

Esophagogastric junction competence was assessed according to the Hill classification (Table 3). Hill grade IV was identified in 37 patients (86.0%), whereas Hill grades III and II were observed in five (11.6%) and one (2.3%) patient, respectively. No patient had Hill grade I.

Hill grade IV predominated in patients with medium-sized and large hiatal hernias, whereas Hill grades II–III were observed mainly in patients with small hiatal hernias.

The association between esophagogastric junction competence, assessed by endoscopic retroflexion, and the maximum size of Cameron lesions was not statistically significant (univariate linear regression) in the studied cohort (*p* = 0.062819), although it approached statistical significance.

#### 3.2.3. Hernia Reducibility

**Figure 2:**
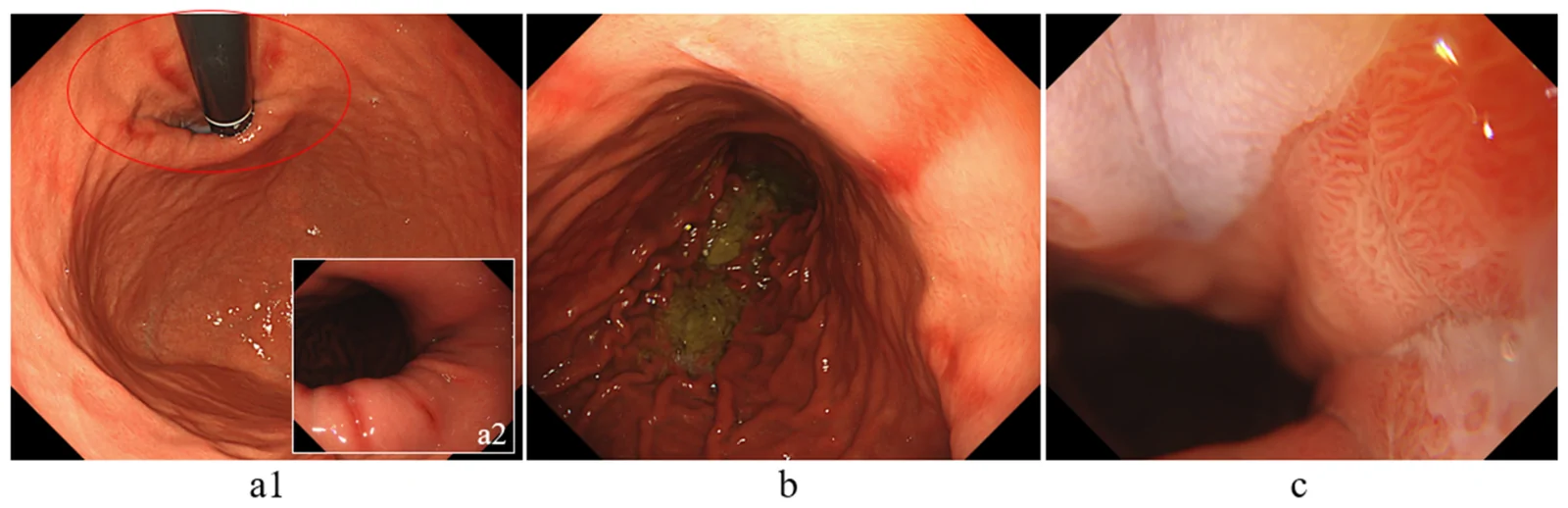
Cameron lesions in small resectable hiatal hernia: (**a1**) retroview from the stomach hightlighting Cameron lesions (red circle) (**a2**) forward endoscopic view, (**b**) forward endoscopic view of hernia neck and (**c**) associated Barrett esophagus C0M2.

The distribution of Cameron lesions according to hernia reducibility is shown in Table 4. Cameron lesions were identified in 17 patients (39.5%) with fixed hiatal hernias, seven (16.3%) with completely reducible hernias, and 19 (44.2%) with partially reducible (mixed) hernias. Representative endoscopic findings in a patient with a small completely reducible hiatal hernia are shown in Figure 2.

#### 3.2.4. Hernia Size

The distribution according to hiatal hernia size is presented in Table 5. Small hiatal hernias (≤30 mm) were identified in nine patients (20.9%), whereas medium-sized (31–50 mm) and large (>50 mm) hiatal hernias were each observed in 17 patients (39.5%). In the studied cohort, the mean hiatal hernia volume was approximately 97.9 cm^3^. The volumetric distribution was heterogeneous, with several large hernias exceeding 250–400 cm^3^, which substantially influenced the arithmetic mean. This quantitative assessment provides a more refined morphological characterization of hiatal hernias and support subsequent analyses exploring potential associations between hernia size and endoscopic findings, including the presence and extent of Cameron lesions.

Cameron lesions were therefore identified across the entire spectrum of hiatal hernia size, including small hiatal hernias.

No significant differences were observed in either the maximum Cameron lesion size (Kruskal–Wallis test, *p* = 0.3108) or the number of Cameron lesions (*p* = 0.9228) among patients with small, medium-sized, and large hiatal hernias (Table 6). Similarly, neither maximum lesion size (*p* = 0.1163) nor lesion number (*p* = 0.1808) differed significantly according to the anatomical type of hiatal hernia (Table 7).

### 3.3. Clinical Presentation and Indications for Endoscopy

The clinical indications for upper gastrointestinal endoscopy are summarized in Table 8. The most common indication was gastroesophageal reflux disease (GERD)-related symptoms, reported in 23 patients (53.4%). Iron-deficiency anemia was the primary indication in nine patients (20.9%), whereas prophylactic or postoperative endoscopic evaluation accounted for eight examinations (18.6%). Overt upper gastrointestinal bleeding was the presenting indication in three patients (7.0%).

Patients referred because of iron-deficiency anemia presented with laboratory findings consistent with chronic blood loss, whereas those with overt upper gastrointestinal bleeding underwent urgent endoscopic evaluation. In patients undergoing prophylactic or postoperative surveillance, Cameron lesions were identified during routine endoscopic assessment in the absence of acute hemorrhagic manifestations.

Overall, Cameron lesions were diagnosed across a broad spectrum of clinical presentations, ranging from incidental findings during elective endoscopy to chronic iron-deficiency anemia and overt upper gastrointestinal bleeding.

### 3.4. Endoscopic Characteristics of Cameron Lesions

The endoscopic characteristics of Cameron lesions are summarized in Table 9. Active lesions were identified in 24 patients (55.8%), cicatricial lesions in 13 (30.2%), and mixed active-cicatricial lesions in 6 (13.9%).

Most patients had multiple Cameron lesions, whereas solitary lesions were observed less frequently. The maximum lesion length ranged from 5 to 35 mm, with a mean of 20.1 ± 6.9 mm. Lesions were typically located on the gastric folds at the level of the diaphragmatic impression and were oriented longitudinally along the long axis of the stomach.

The most common associated endoscopic findings included fibrinous exudate, mucosal erythema, edema, and mucosal friability. In patients with completely reducible hiatal hernias, lesion visibility occasionally depended on adequate insufflation and careful examination of the diaphragmatic impression.

Correlation analyses demonstrated no significant association between hiatal hernia height and the number of Cameron lesions (Spearman’s ρ = −0.020, *p* = 0.901) or between estimated hiatal hernia volume and lesion number (ρ = −0.056, *p* = 0.723). Likewise, maximum lesion length was not significantly correlated with hiatal hernia height (Pearson’s *r* = 0.243, *p* = 0.116) or estimated hernia volume (r = 0.121, *p* = 0.438). Linear regression analysis similarly showed no significant relationship between hiatal hernia dimensions and maximum Cameron lesion length.

### 3.5. Stigmata of Bleeding and Hemorrhagic Manifestations

The hemorrhagic manifestations of Cameron lesions are summarized in Table 10. According to the Forrest classification, 24 patients (55.81%) had Forrest III lesions without endoscopic signs of recent bleeding, whereas 19 patients (44.18%) exhibited active or recent hemorrhagic stigmata. Forrest Ia, Ib, IIa, IIb, and IIc lesions were identified in 1 (2.3%), 8 (18.6%), 2 (4.65%), 5 (11.6%), and 3 (7.0%) patients, respectively.

Endoscopic evidence of active or recent bleeding included active arterial or oozing hemorrhage, adherent clots, non-bleeding visible vessels, fresh blood within the stomach, blood streaks over the lesion surface, and contact bleeding during endoscopic examination. Hemosiderin pigmentation, consistent with previous bleeding episodes, was also observed in a subset of patients.

Patients with overt upper gastrointestinal bleeding generally presented with Forrest Ia–IIb lesions, whereas patients investigated for chronic iron-deficiency anemia more frequently had Forrest IIc or III lesions. Endoscopic hemostatic therapy was performed when clinically indicated in patients with active bleeding or high-risk stigmata.

### 3.6. Associated Upper Gastrointestinal Pathology

Associated upper gastrointestinal disorders are summarized in Table 11. Reflux esophagitis was the most frequent associated finding, documented in 23 patients (53.4%) and classified according to the Los Angeles and Savary–Miller systems. Barrett’s esophagus, confirmed histologically as specialized intestinal metaplasia, was identified in 7 patients (16.3%).

Erosive-hemorrhagic or macular-erosive gastritis was present in 18 patients (41.9%), duodenogastric bile reflux in 10 patients (23.3%), and erosive duodenitis in 7 patients (16.3%).

Additional upper gastrointestinal abnormalities were identified in a small number of patients and included Lyall-type esophageal ulcers, Savary-type esophageal ulcers, gastric submucosal nodules, and a duodenal adenoma involving the major papilla. Detailed descriptions of these uncommon presentations are provided in Section 3.9 (Illustrative Clinical Cases).

### 3.7. Helicobacter pylori Status

*Helicobacter pylori* status was assessed using the rapid urease test in 35 of the 43 patients (81.4%). Eleven patients tested positive, corresponding to a positivity rate of 31.4% among those tested and 25.6% of the entire study cohort, whereas 24 patients (68.6%) tested negative. No statistically significant association was observed between *H. pylori* infection and the endoscopic characteristics or hemorrhagic manifestations of Cameron lesions.

### 3.8. Postoperative Outcomes in Patients with Previous Fundoplication

Three patients (7.0%) had a history of anti-reflux fundoplication performed for hiatal hernia (Table 12). Cameron lesions had not been documented before surgery in any of these patients. During postoperative follow-up, all three developed recurrent hiatal hernia associated with newly diagnosed Cameron lesions.

One patient developed a recurrent type I (axial) hiatal hernia following disruption of a laparoscopic Toupet fundoplication. The remaining two patients presented with recurrent type III (mixed) hiatal hernias caused by migration of the fundoplication into the mediastinum after failure of the hiatal repair.

**Figure 3:**
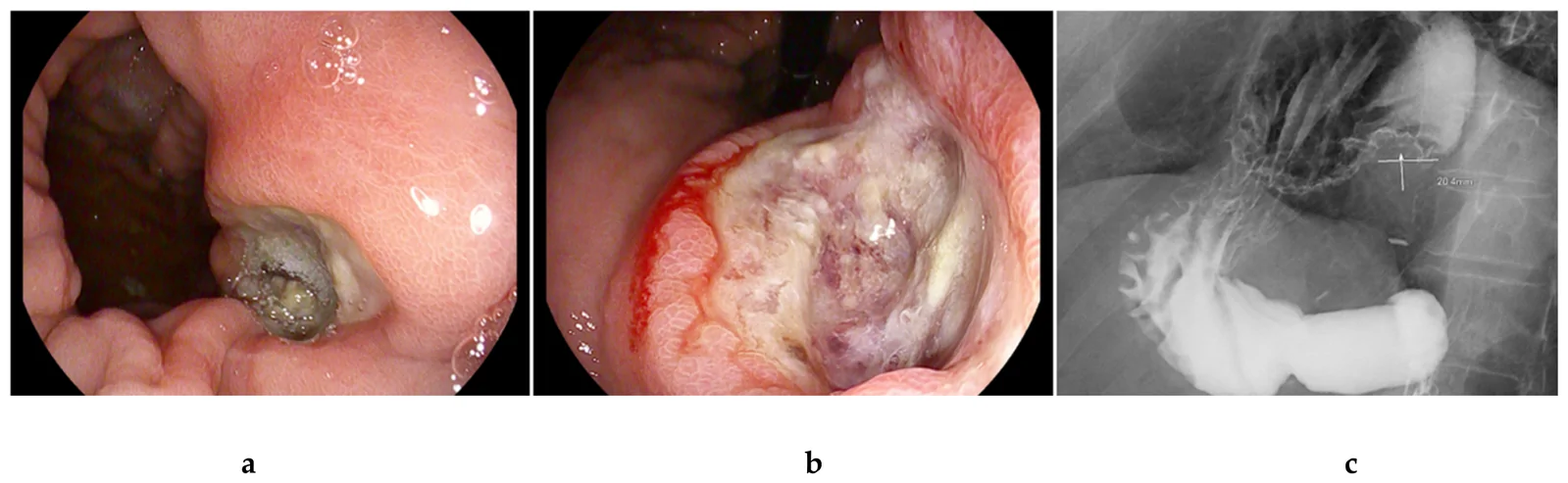
Untreated Cameron lesion, which evolved to malignancy, histologically high-grade dysplasia (HGD) in the biopsy from the edges of the lesion: (**a**) Cameron lesion on the neck of the hiatal hernia in forward-view, (**b**) suspicious malignant appearance of the Cameron lesion in retroview from the stomach and (**c**) hiatal hernia and ulcerative niche on barium fluoroscopy (arrow). The coexistence of the two lesions (Cameron ulceration and malignancy) should not lead to premature conclusions regarding a direct causal relationship.

All three patients had active Cameron lesions with high-risk hemorrhagic stigmata (Forrest Ia), intermediate-risk (Forrest IIa) and low-risk (Forrest III) documented during postoperative endoscopic evaluation. The postoperative endoscopic findings are illustrated in Figure 3.

### 3.9. Illustrative Clinical Cases

Several patients presented with uncommon clinical and endoscopic features that illustrate the broad spectrum of Cameron lesion presentation observed in the present series.

Small completely reducible hiatal hernias. Two patients, aged 38 and 40 years, had small (<3 cm), completely reducible type I hiatal hernias associated with multiple Cameron lesions, demonstrating that these lesions may also occur in small, fully reducible hernias.

Post-fundoplication recurrence. A 59-year-old woman developed recurrent type I hiatal hernia following laparoscopic Toupet fundoplication. Cameron lesions, which had not been documented before surgery, were identified during postoperative endoscopic evaluation after hernia recurrence.

Young age at diagnosis. The youngest patient in the series was a 31-year-old man with a completely reducible type I hiatal hernia, multiple Cameron lesions, Los Angeles grade B reflux esophagitis, and Barrett’s esophagus (Prague classification C0M2).

Histological changes. A 77-year-old woman with a recurrent type III hiatal hernia developed progressive morphological changes in a chronic Cameron ulcer during endoscopic follow-up. Targeted biopsies demonstrated high-grade dysplasia (Figure 3).

Confirmed source of upper gastrointestinal bleeding. The oldest patient in the series, an 83-year-old woman with a large type I hiatal hernia, had multiple Cameron ulcers that were endoscopically confirmed as the source of overt upper gastrointestinal bleeding.

## 4. Discussion

### 4.1. Main Findings

The present study represents the first comprehensive Moldavian case series of patients with Cameron lesions and provides a detailed analysis of their demographic characteristics, hiatal hernia anatomy, clinical presentation, endoscopic morphology, hemorrhagic manifestations, associated upper gastrointestinal disorders, *Helicobacter pylori* status, and postoperative outcomes following anti-reflux surgery [27,28]. The principal findings of this study are as follows.

First, Cameron lesions were identified across the entire spectrum of hiatal hernia size, including small, completely reducible hiatal hernias, indicating that these lesions are not confined to large paraesophageal or mixed hernias. Second, neither the maximum lesion size nor the number of Cameron lesions showed a significant association with hiatal hernia size or anatomical type, suggesting that factors other than hernia dimensions may contribute to lesion development. Third, nearly half of the patients exhibited active or recent hemorrhagic stigmata according to the Forrest classification, highlighting the clinical relevance of these lesions as a source of upper gastrointestinal bleeding. Fourth, Cameron lesions developed in patients with recurrent hiatal hernia following failed fundoplication, supporting the importance of postoperative anatomical integrity. Finally, *H. pylori* infection showed no apparent association with the endoscopic characteristics or hemorrhagic manifestations of Cameron lesions.

These findings expand the current understanding of Cameron lesions by emphasizing their heterogeneous clinical and endoscopic presentation and underscore the importance of careful endoscopic examination in patients with hiatal hernias, irrespective of hernia size or previous surgical treatment [27].

### 4.2. Hiatal Hernia Characteristics and the Risk of Cameron Lesions

Cameron lesions have traditionally been regarded as a complication of large hiatal hernias, particularly paraesophageal and mixed hernias, where repetitive mechanical trauma at the diaphragmatic hiatus is considered the principal pathogenic mechanism. Most published studies have therefore emphasized lesion occurrence in patients with large hernias associated with substantial cranio-caudal movement and chronic mucosal compression during respiration and swallowing. Similar observations have been reported by Cameron and Higgins, Weston, Moskovitz, and subsequent investigators [1,26,28].

Our findings partially support this concept while also expanding the current understanding of Cameron lesions. Although medium-sized and large hiatal hernias accounted for most cases in our cohort, Cameron lesions were also identified in small, completely reducible type I hiatal hernias. This observation indicates that large hernia size is not an absolute prerequisite for lesion development and suggests that careful endoscopic inspection should not be limited to patients with advanced hiatal hernias.

A significant association was observed between anatomical hiatal hernia type and hernia size, with type III hernias predominating among large hernias and type I hernias occurring mainly in small defects. However, neither the maximum Cameron lesion size nor the number of lesions was significantly associated with hernia size or anatomical classification. These findings suggest that, although hernia anatomy influences the anatomical setting in which Cameron lesions develop, it does not appear to determine lesion extent or multiplicity. Additional factors, including repetitive mucosal trauma, local ischemia, gastric mobility, and individual susceptibility, may also contribute to lesion formation and progression [29].

An additional observation was the predominance of Hill grade IV esophagogastric junction incompetence among affected patients. This finding is consistent with the severe disruption of the anti-reflux barrier commonly observed in patients with clinically significant hiatal hernias. Nevertheless, Cameron lesions were also identified in patients with less advanced junctional incompetence, indicating that complete loss of esophagogastric junction competence is not essential for their development.

### 4.3. Clinical Presentation

The clinical presentation of Cameron lesions remains highly heterogeneous, ranging from incidental endoscopic findings to chronic iron-deficiency anemia and life-threatening upper gastrointestinal bleeding. This broad clinical spectrum has been consistently reported in the literature and reflects differences in lesion activity, chronicity, and bleeding severity rather than the anatomical characteristics of the hiatal hernia alone [10].

In the present series, gastroesophageal reflux disease (GERD)-related symptoms represented the most frequent indication for upper gastrointestinal endoscopy, whereas iron-deficiency anemia and overt upper gastrointestinal bleeding accounted for approximately one-quarter of all examinations. In addition, Cameron lesions were identified during prophylactic or postoperative endoscopic surveillance in patients without acute hemorrhagic manifestations. These findings indicate that Cameron lesions should not be regarded solely as a cause of gastrointestinal bleeding but also as an incidental or unsuspected endoscopic finding in patients undergoing evaluation for other indications.

Chronic occult blood loss resulting in iron-deficiency anemia remains one of the most characteristic clinical manifestations of Cameron lesions and is frequently the reason for diagnostic investigation [30]. However, our findings also confirm that these lesions may present with overt upper gastrointestinal bleeding when active hemorrhagic stigmata are present. Consequently, Cameron lesions should be included in the differential diagnosis of both unexplained iron-deficiency anemia and upper gastrointestinal bleeding in patients with hiatal hernias, regardless of hernia size.

An additional clinically relevant observation was the identification of Cameron lesions during routine postoperative surveillance after anti-reflux surgery. Although these patients were asymptomatic with respect to acute bleeding, recurrent hiatal hernia was accompanied by the appearance of Cameron lesions, emphasizing the importance of meticulous endoscopic evaluation whenever anatomical recurrence is suspected.

### 4.4. Endoscopic Characteristics and Bleeding Patterns

The endoscopic appearance of Cameron lesions is highly variable, reflecting different stages of mucosal injury and healing.

The performance of equipment is just as important as the endoscopist’s experience when evaluating such pathology.

In the present study, active lesions predominated, whereas cicatricial and mixed lesions accounted for a smaller proportion of cases. This distribution suggests that Cameron lesions represent a dynamic process characterized by repeated episodes of mucosal injury followed by partial healing, rather than a static pathological entity. Similar observations have been reported in previous endoscopic series.

Cameron lesions were typically located on the crests of the gastric folds at the level of the diaphragmatic impression and were oriented longitudinally along the gastric axis. These characteristic endoscopic features support the concept that repetitive mechanical trauma and localized ischemia at the diaphragmatic hiatus play complementary roles in lesion development [31]. However, the absence of significant correlations between hiatal hernia dimensions and either lesion size or lesion number indicates that factors other than hernia size alone are involved in determining lesion morphology.

Nearly half of the patients exhibited active or recent hemorrhagic stigmata according to the Forrest classification, confirming that Cameron lesions are clinically relevant sources of upper gastrointestinal bleeding. The use of the Forrest classification in the present study allowed a standardized assessment of bleeding activity and demonstrated that Cameron lesions encompass a broad spectrum of hemorrhagic severity, ranging from chronic inactive ulcers to actively bleeding lesions requiring endoscopic treatment. Although the Forrest classification is widely used for peptic ulcer bleeding, it has rarely been applied systematically to Cameron lesions, representing a particular strength of the present study.

An additional practical observation concerns lesion detection. In several patients, particularly those with completely reducible hiatal hernias, adequate insufflation and careful inspection of the diaphragmatic impression were necessary to identify Cameron lesions. These findings emphasize the importance of a meticulous endoscopic examination of the hiatal region, especially in patients with small or reducible hiatal hernias, in whom these lesions may easily be overlooked.

### 4.5. Hemorrhagic Manifestations

Hemorrhage represents the most clinically significant complication of Cameron lesions and may range from chronic occult blood loss to acute upper gastrointestinal bleeding. Although chronic iron-deficiency anemia remains the most frequently reported clinical consequence, active bleeding is increasingly recognized owing to improved endoscopic awareness and careful inspection of the diaphragmatic impression during upper gastrointestinal endoscopy. Similar observations have been reported in previous clinical series [32].

In the present study, nearly half of the patients demonstrated active or recent hemorrhagic stigmata according to the Forrest classification, confirming that Cameron lesions should be regarded as clinically relevant bleeding lesions rather than incidental mucosal abnormalities. The wide spectrum of Forrest stages observed in our cohort reflects different phases of mucosal injury and healing and illustrates the dynamic nature of these lesions.

The coexistence of chronic iron-deficiency anemia in some patients and overt upper gastrointestinal bleeding in others further supports the concept that Cameron lesions represent a continuum of hemorrhagic activity rather than distinct clinical entities. Chronic superficial mucosal trauma may result in persistent occult blood loss, whereas repeated mechanical injury or progressive ulceration may eventually expose submucosal vessels and produce clinically significant hemorrhage. This mechanism is consistent with the widely accepted pathophysiological model of repetitive mechanical stress combined with localized ischemia at the diaphragmatic hiatus.

These findings emphasize that the absence of massive hemorrhage does not exclude clinically important blood loss. Consequently, Cameron lesions should be considered in patients with otherwise unexplained iron-deficiency anemia as well as in those presenting with overt upper gastrointestinal bleeding, particularly when a hiatal hernia is identified during endoscopic evaluation.

### 4.6. Helicobacter pylori Infection

The potential contribution of *Helicobacter pylori* infection to the pathogenesis of Cameron lesions remains uncertain. Unlike peptic ulcer disease, Cameron lesions are generally considered to result predominantly from repetitive mechanical trauma and localized ischemia at the level of the diaphragmatic hiatus rather than from infectious or acid-related mechanisms. Previous studies have reported inconsistent findings regarding the prevalence and possible role of *H. pylori* in affected patients, and no causal relationship has been established [26,29].

In the present study, approximately one-third of the tested patients were positive for *H. pylori*. However, no apparent association was observed between *H. pylori* infection and the endoscopic characteristics or hemorrhagic manifestations of Cameron lesions. These findings support the view that *H. pylori* is unlikely to represent a major determinant of lesion development or bleeding risk in this clinical setting, although its identification remains clinically relevant because of its established role in other gastroduodenal disorders and the potential indication for eradication therapy according to current clinical guidelines. In our cohort, *H. pylori* status was not significantly associated with lesion morphology, lesion size, lesion number, or hemorrhagic activity.

### 4.7. Cameron Lesions Following Failed Fundoplication

An important observation in the present study was the identification of Cameron lesions exclusively in patients with recurrent hiatal hernia following previous anti-reflux surgery. None of these patients had documented Cameron lesions before fundoplication, whereas all developed recurrent hiatal hernias that were subsequently associated with newly detected Cameron lesions. Although the number of cases was limited, this finding suggests that restoration of the anatomical conditions predisposing to repetitive mucosal trauma may contribute to the development of Cameron lesions after surgical failure.

The recurrence of hiatal hernia following fundoplication has been recognized as one of the principal causes of recurrent gastroesophageal reflux symptoms and anatomical failure after anti-reflux surgery [33]. Re-establishment of the hernia recreates the mechanical environment responsible for repetitive compression of the gastric mucosa at the diaphragmatic hiatus, thereby providing a plausible explanation for the appearance of Cameron lesions in these patients.

Although our observations are based on a small number of patients, they emphasize the importance of careful endoscopic evaluation in individuals presenting with recurrent hiatal hernia after fundoplication. In addition to assessing recurrent reflux esophagitis and the integrity of the fundoplication, endoscopists should also inspect the diaphragmatic impression for Cameron lesions, particularly when patients present with iron-deficiency anemia or upper gastrointestinal bleeding. Further multicenter studies are warranted to determine the incidence and clinical significance of Cameron lesions in this postoperative setting. To our knowledge, reports specifically describing Cameron lesions following recurrent hiatal hernia after fundoplication remain scarce.

### 4.8. Clinical Implications

The findings of the present study have several practical implications for the endoscopic evaluation and management of patients with hiatal hernias. First, Cameron lesions should not be regarded as an exclusive complication of large hiatal hernias, as they may also occur in small, completely reducible defects. Consequently, careful inspection of the diaphragmatic impression should be incorporated into every upper gastrointestinal endoscopic examination in patients with hiatal hernias, irrespective of hernia size or the primary indication for endoscopy.

Second, meticulous endoscopic technique is essential for lesion detection. Adequate gastric insufflation, thorough examination of the crests of the gastric folds at the level of the diaphragmatic hiatus, and repeated inspection during both insertion and withdrawal of the endoscope may improve the identification of Cameron lesions. This is particularly important in patients with reducible hiatal hernias, in whom these lesions may be transiently concealed and therefore easily overlooked.

Third, Cameron lesions should be routinely considered in the differential diagnosis of both unexplained iron-deficiency anemia and overt upper gastrointestinal bleeding in patients with hiatal hernias. Because these lesions may be subtle or overlooked during routine examination, their recognition requires a high index of suspicion. Failure to identify Cameron lesions may delay appropriate treatment, resulting in persistent anemia, recurrent bleeding episodes, repeated diagnostic investigations, and avoidable healthcare utilization.

The systematic assessment of bleeding activity using the Forrest classification may further assist in clinical decision-making. Recognition of active or recent hemorrhagic stigmata allows timely endoscopic hemostatic therapy and facilitates risk stratification according to established principles for the management of non-variceal upper gastrointestinal bleeding. Patients with recurrent hemorrhage, persistent iron-deficiency anemia despite optimized medical therapy, or recurrent hiatal hernia following previous surgical repair should undergo multidisciplinary evaluation to determine whether definitive surgical correction is indicated.

Finally, the identification of Cameron lesions in patients with recurrent hiatal hernia after fundoplication expands the spectrum of clinical settings in which these lesions should be actively sought. Endoscopic evaluation of patients with suspected anatomical recurrence after anti-reflux surgery should therefore include careful assessment of the diaphragmatic impression in addition to the conventional evaluation of fundoplication integrity and reflux-related mucosal injury.

Overall, our findings emphasize that increased awareness of Cameron lesions, combined with meticulous endoscopic examination and appropriate therapeutic management, may improve diagnostic accuracy, reduce missed lesions, and contribute to better clinical outcomes in patients with hiatal hernias.

### 4.9. Strengths and Limitations

The present study has several strengths. It provides a comprehensive single-center analysis of patients with Cameron lesions, including detailed demographic, clinical, and endoscopic characterization. Beyond the conventional description of lesion morphology, we systematically evaluated hiatal hernia anatomy, esophagogastric junction competence according to the Hill classification, hernia reducibility, hemorrhagic activity using the Forrest classification, *Helicobacter pylori* status, and postoperative findings in patients with recurrent hiatal hernia following fundoplication. Furthermore, the integration of detailed endoscopic observations with statistical analyses allowed a comprehensive assessment of the relationships between hiatal hernia characteristics and Cameron lesion morphology. The inclusion of patients with postoperative recurrent hiatal hernia and newly developed Cameron lesions also provides additional clinical information on a relatively underreported aspect of this condition.

Nevertheless, several limitations should be acknowledged. First, the retrospective design may have introduced selection and information bias. Second, the study was conducted at a single tertiary referral center, which may limit the generalizability of the findings to other populations and clinical settings. Third, although relatively large for this uncommon condition, the sample size remained limited, reducing the statistical power of subgroup analyses. In addition, *H. pylori* testing was performed only when clinically indicated and technically feasible, reflecting routine clinical practice rather than a predefined study protocol. Finally, long-term follow-up was not available for all patients, precluding a comprehensive assessment of lesion recurrence, long-term bleeding outcomes, and the effectiveness of different therapeutic strategies.

Despite these limitations, the present study expands the current understanding of Cameron lesions by providing a comprehensive evaluation of their clinical presentation, endoscopic characteristics, hemorrhagic manifestations, and relationship with hiatal hernia anatomy. The findings emphasize the importance of meticulous endoscopic examination in patients with hiatal hernias and provide a basis for future prospective multicenter studies aimed at validating risk factors, optimizing diagnostic strategies, and defining the role of endoscopic and surgical management in selected patients.

## 5. Conclusions

Cameron lesions represent a clinically important but frequently underrecognized complication of hiatal hernias, exhibiting a broad spectrum of endoscopic and clinical manifestations, ranging from incidental findings to chronic iron-deficiency anemia and overt upper gastrointestinal bleeding. Their heterogeneous presentation highlights the need for increased awareness among endoscopists and clinicians involved in the evaluation of patients with hiatal hernias.

Our findings demonstrate that Cameron lesions may occur throughout the entire spectrum of hiatal hernia anatomy, including small, completely reducible hernias, and that lesion size and multiplicity are not significantly associated with hernia size or anatomical type. These observations suggest that mechanisms beyond hernia dimensions alone contribute to lesion development and support the concept of a multifactorial pathogenesis involving repetitive mechanical trauma and localized mucosal ischemia.

Meticulous endoscopic examination of the diaphragmatic impression, supported by adequate insufflation and systematic inspection of the gastric folds, is essential for accurate lesion detection. The routine assessment of hemorrhagic activity, including the application of the Forrest classification when appropriate, may improve recognition of clinically significant bleeding and facilitate appropriate therapeutic decision-making.

The identification of Cameron lesions in patients with recurrent hiatal hernia following failed fundoplication further expands the clinical spectrum of this condition and underscores the importance of careful postoperative endoscopic surveillance. In contrast, *Helicobacter pylori* infection did not appear to play a major role in the development or hemorrhagic behavior of Cameron lesions in our cohort.

Overall, this study provides a comprehensive clinical and endoscopic characterization of Cameron lesions and reinforces the importance of their systematic evaluation during upper gastrointestinal endoscopy. Improved recognition of these lesions may enhance diagnostic accuracy, reduce missed diagnoses, and facilitate timely medical, endoscopic, or surgical management. Future prospective multicenter studies are warranted to validate these findings, further elucidate the natural history of Cameron lesions, and optimize evidence-based diagnostic and therapeutic strategies.

## Figures and Tables

**Table 1:** Demographic characteristics of the study cohort.

Parameter	Value	Percent (%)
Total unique patients	43	100%
Women	28	65.1%
Men	15	34.9%
Mean age ± SD (years)	60.4 ± 12.7	—
Age range (years)	31–83	—
Patients with follow-up visits	40	93%
Patients with previous fundoplication	3	7.0%
Collection period	2017–2026	9 years

**Table 2:** Anatomical classification.

Type of Hiatal Hernia	No	Percent (%)
Type I—axial sliding	14	32.6%
Type II—paraesophageal (rolling)	15	34.8%
Type III—mixed (slip + roll)	14	32.6%
Type IV—with involvement of other organs	0	0%
Total	43	100%

**Table 3:** Hill Classification.

GEJ Incompetence	HH Small (≤30 mm)	HH Medium (31–50 mm)	HH Big (>50 mm)	No	Percent (%)
Hill I	0	0	0	0	0%
Hill II	1	0	0	1	2.3%
Hill III	3	2	0	5	11.6%
Hill IV	5	15	17	37	86.0%
Total	9	17	17	43	100%

**Table 4:** The distribution of Cameron lesions according to hernia reducibility.

Hiatal Hernia Reducibility	No	Percent (%)
Fixed hernias (non-reducible)	17	39.5%
Completely reducible hernias	7	16.3%
Partially reducible hernias	19	44.2%
Total	43	100%

**Table 5:** The distribution of Cameron lesions according to hiatal hernia size.

Hiatal Hernia Reducibility	No	Percent (%)
Small hernias (≤30 mm)	9	20.9%
Median hernias (31–50 mm)	17	39.5%
Big hernias (>50 mm)	17	39.5%
Total	43	100%

**Table 6:** Statistical associations between hiatal hernia size and number and size of Cameron lesions.

Median [Q1, Q3]	Large HH (*n* = 17)	Medium-Size HH (*n* = 17)	Small HH (*n* = 9)	*p*_Value
Maximum size of the Cameron lesion	20.0 [17.0, 27.5]	18.0 [15.0, 21.5]	20.0 [11.5, 25.0]	0.310845 (Kruskal–Wallis H)
No of Cameron lesions	4.0 [1.5, 5.0]	4.0 [3.0, 4.5]	4.0 [3.0, 5.0]	0.922832 (Kruskal–Wallis H)

**Table 7:** Statistical associations between hiatal hernia type and number and size of Cameron lesions.

Median [Q1, Q3]	HH Type I (*n* = 14)	HH Type II (*n* = 15)	HH Type III (*n* = 14)	*p*_Value
Maximum size of the Cameron lesion	24.0 [19.5, 25.0]	18.0 [15.0, 23.0]	18.0 [15.0, 22.5]	0.116268 (Kruskal–Wallis H)
No of Cameron lesions	4.0 [1.0, 4.25]	4.0 [2.0, 5.0]	4.0 [4.0, 5.0]	0.180766 (Kruskal–Wallis H)

**Table 8:** Distribution of indications for upper digestive endoscopy.

Endoscopy Indication	No	Percent (%)
GERD symptoms/epigastric pain	23	53.4%
Confirmed or suspected iron deficiency anemia	9	20.9%
Asymptomatic prophylactic endoscopic control	5	11.6%
Postoperative endoscopic control (post-symptomatic FP)	3	7.0%
Manifest upper digestive bleeding	3	7.0%
Total	43	100%

**Table 9:** Endoscopic features of Cameron lesions.

Endoscopic Feature	No	Percent (%)
Active Cameron ulcers (A1, A2, H1, H2 Sakita-Miwa)	24	55.8%
Cicatricial sequelae (S1, S2 Sakita-Miwa)	13	30.2%
Mixed picture (active ulceration + scarring)	6	13.9%
Multiple lesions (≥2 on the same examination)	38	88.4%

**Table 10:** Hemorrhagic manifestations of Cameron lesions and Forrest classification.

Forrest Classification	No	Percent (%)
Forrest Ia (pulsatile active bleeding)	1	2.3%
Forrest Ib (non-pulsatile active bleeding)	8	18.6%
Forrest IIa (visible non-bleeding vessel)	2	4.65%
Forrest IIb (adherent clot)	5	11.6%
Forrest IIc (hemosiderin)	3	7.0%
Forrest III (potentially hemorrhagic, currently non-hemorrhagic)	24	55.81%

**Table 11:** Associated upper gastrointestinal pathology in patients with Cameron lesions.

Associated Pathology	No	Percent (%)
Reflux esophagitis	23	53.4%
Barrett’s esophagus	7	16.3%
Erosive/macular gastritis	18	41.9%
Duodeno-gastric bile reflux	10	23.3%
Erosive duodenitis	7	16.3%
Lyall-type esophageal ulcer	1	2.32%
Savary-type esophageal ulcer	3	6.97%
Gastric submucosal nodules	2	4.65%
Major papilla adenoma	1	2.3%

**Table 12:** Postoperative characteristics of patients with recurrent Cameron lesions after fundoplication.

Variable	Patient 1	Patient 2	Patient 3
Age/Sex	59 F	68 F	60 M
Fundoplication type	Toupet	Nissen	Nissen
Recurrent hernia type	I	III	III
Mechanism of recurrence	Disruption	Migration	Migration
Cameron lesions before surgery	No	No	No
Cameron lesions after recurrence	Yes	Yes	Yes
Forrest classification	III	Ia	IIa

## Data Availability

The data are available from the corresponding author upon reasonable request.

## Abbreviations

GERD	Gastroesophageal reflux disease
